# Probiotics, prebiotics, and synbiotics in chronic constipation: Outstanding aspects to be considered for the current evidence

**DOI:** 10.3389/fnut.2022.935830

**Published:** 2022-12-08

**Authors:** Maísa Miranda Araújo, Patrícia Borges Botelho

**Affiliations:** Department of Nutrition, Faculty of Health Science, University of Brasília, Brasília, Brazil

**Keywords:** probiotic, constipation, gastrointestinal symptoms, defecation frequency, prebiotic, synbiotic, review

## Abstract

This integrative aimed to evaluate the effects and the potential mechanism of action of prebiotics, probiotics, and synbiotics on constipation-associated gastrointestinal symptoms and to identify issues that still need to be answered. A literature search was performed in the PubMed database. Animal models (*n* = 23) and clinical trials (*n* = 39) were included. In animal studies, prebiotic, probiotic, and synbiotic supplementation showed a decreased colonic transit time (CTT) and an increase in the number and water content of feces. In humans, inulin is shown to be the most promising prebiotic, while *B. lactis* and *L. casei Shirota* probiotics were shown to increase defecation frequency, the latter strain being more effective in improving stool consistency and constipation symptoms. Overall, synbiotics seem to reduce CTT, increase defecation frequency, and improve stool consistency with a controversial effect on the improvement of constipation symptoms. Moreover, some aspects of probiotic use in constipation-related outcomes remain unanswered, such as the best dose, duration, time of consumption (before, during, or after meals), and matrices, as well as their effect and mechanisms on the regulation of inflammation in patients with constipation, on polymorphisms associated with constipation, and on the management of constipation *via* 5-HT. Thus, more high-quality randomized control trials (RCTs) evaluating these lacking aspects are necessary to provide safe conclusions about their effectiveness in managing intestinal constipation.

## Introduction

Chronic constipation (CC) is a common disorder characterized by difficult stool passage and/or infrequent bowel movements, at least for a period of 3 months (1). Patients suffering from CC can present non-specific symptoms, such as hard stools, abdominal discomfort and/or distention, bloating, and flatulencies (2). This disorder has a high prevalence, affecting about 12–14% of the global population, with a higher prevalence among women and the elderly (3, 4).

The diagnosis of CC is based on the patient’s clinical history and physical and proctology examination (5). To standardize the diagnosis of CC, one of the criteria proposed and most widely used is the Rome criteria, which distinguish primary and secondary constipation types from irritable bowel syndrome (IBS) (2). The primary type is idiopathic, and the secondary is a result of medication use, a disease, or even dietary (6).

The high prevalence and chronicity of this condition highlight the importance of early assertive intervention to improve symptoms and the patient’s quality of life (QoL) and prevent future high health costs (7). There are currently several treatments used to manage CC, including medications (i.e., laxatives and prokinetic agents), behavior change (i.e., physical activity), and dietary interventions, such as an increase in water and dietary fiber intake (8). Among dietary interventions, prebiotic, probiotic, and synbiotic supplementations have been increasingly investigated as potential treatment alternatives for CC (9–11).

Despite the growing number of studies evaluating the effect of prebiotics, probiotics, and synbiotics on CC, so far, they have reported controversial effects on constipation-related outcomes and with high heterogeneity, due to differences in the protocols used (12, 13). Therefore, this review aims to evaluate the effects and the potential mechanism of action of prebiotics, probiotics, and synbiotics on gastrointestinal symptoms in constipation, carefully considering the variations of protocols (i.e., probiotic strains, dose, duration of intervention, vehicle, and form of administration), and to identify those issues that have not yet been answered, thus stimulating studies with more appropriate and robust experimental designs.

## Methods

### Integrative review and search strategy

A literature review was performed in the Medline database (*via* PubMed). The following search strategy was used in database: (constipation) AND (probiotic OR prebiotic OR synbiotic) AND (“Digestive symptoms” OR “Digestive symptom” OR “Gastrointestinal symptoms” OR Dyschezia OR “Colonic Inertia” OR “Colonic Transit Time” OR “Whole Gut Transit” OR “Bowel Movement” OR “Bowel Movements” OR Bloating OR Flatus OR Flatulence OR Feces OR “Stool Frequency” OR “Stool Consistency” OR “Low Defecation Frequency” OR “Defecation Frequency” OR “Gastrointestinal Transit” OR “Gastrointestinal Transits” OR “Gastrointestinal, Motilities” OR “Gastrointestinal, Motility” OR “Motility, Gastrointestinal” OR “Gastrointestinal Motilities” OR “Intestinal Motility” OR “Intestinal Motilities” OR “Motility, Intestinal” OR “ROME III” OR “ROME III criteria” OR “ROME IV” OR “ROME IV criteria” OR diary OR “Bristol Stool Form” OR “Bristol Stool Chart” OR “Bristol stool form scale” OR “Gastrointestinal symptoms questionnaire” OR “Gastrointestinal symptom questionnaire” OR “Intestinal symptom questionnaire”). The duplicates were removed, and the screening procedure was conducted in Rayyan software.

### Eligible criteria

We included research in animal models (*n* = 23) and clinical trials (*n* = 39) that evaluated the effect of probiotics or prebiotics or synbiotics on constipation-related symptoms. Integrative (*n* = 83) and systematic review (*n* = 18) studies were retrieved on literature search, and when relevant, they were included to define and discuss the mechanisms of action of those dietary supplementations in the evaluated outcomes.

## Results

To better understand the outstanding factors to be regarded for probiotic, prebiotic, and symbiotic use on constipation, we discuss what chronic constipation is, their risk factors, and treatment and then understand how these compounds could have better performance and what are the factors that can contribute to this.

### Chronic constipation

Chronic constipation is commonly divided into two categories: primary and secondary. The primary or functional constipation can be classified as follows: (i) normal transit constipation, (ii) slow transit constipation, (iii) anorectal dysfunction, and (iv) combined causes (slow transit constipation and pelvic floor dysfunction) (14). In addition, secondary CC can occur as a result of medication (opioids or antihypertensive agents) or diseases (hypothyroidism or Parkinson’s disease, colorectal cancer, or diverticular stricture) (6).

The pathophysiology of CC is multifactorial and not well understood. The mechanisms elucidated for CC involved an imbalance or dysfunction for some components such as the following: enteric nervous system (ENS), a neural gastrointestinal (GI) neural network composed of enteric neurons and enteric glial cells, which can communicate to diverse cell types as enterochromaffin, interstitial, and mast cells, stimulating intestinal peristalsis and colonic motility; autonomic nervous system (ANS), which can inhibit intestinal motility *via* sympathetic nerve or excite by vagus nerve *via* parasympathetic nerve; central nervous system (CNS), which can induce gut motility through ANS regulation and by hormonal pathways, including the hypothalamus–pituitary–adrenal axis and hormones of the neuroendocrine stress response (15).

Chronic constipation may also be related to the dysregulation of other components such as intestinal ion channels, which play a role in maintaining the balance of intestinal absorption and secretion and enhancing gastrointestinal transit (GIT) and fecal excretion; aquaporins (APQS), by the transmembrane transport of water molecules in the intestine; endocrine signaling, which can regulate gut peristalsis by GI hormones as motilin, gastrin, melatonin, and somatostatin; microbiota composition, with the reduction in short-chain fatty acids (SCFAs) producing bacteria and increase in lipopolysaccharide (LPS) production, consequently reducing gut peristalsis; as well as the dietary and behavioral factors (15).

### Prevalence, risk factor, and diagnostics of CC

The prevalence of CC varies among studies according to the definition used and among countries. Overall, the average prevalence of chronic idiopathic constipation in adults worldwide has been estimated at 14% (95% CI: 12–17%) after the evaluation of 41 studies (*n* = 261,040 participants) (4). The main risk factors of CC supported by evidence are age, gender, and socioeconomic status (4).

It is well known that CC prevalence increases with age, due to the lack of bowel movements, inadequate fiber and fluid intake, lower physical activity, illness, and higher intake of medicines for the elderly. In the elderly, constipation in women is almost 2-fold more frequent than in men (17.4–9.2%) (4). This could be explained by the fluctuations in female sex hormones during pre- and post-menopausal periods, a higher chance of damage to pelvic floor muscles during childbirth, and the fact that women are more likely to seek healthcare for constipation (16).

Beyond female gender, advanced age and low socioeconomic status, the use of medications, and dietary and lifestyle factors are also risk factors described as associated with constipation. Medications such as opioids, calcium channel blockers, and antidepressants can alter gut motility by different mechanisms (reduction in propulsive contractions, decrease in water in the bowel, and colonic transit time). With regards to the dietary factors, the high-fiber diet (OR = 0.33, 95% CI: 0.15–0.75) and high-water intake of 0.35 (95% CI: 0.14–0.87) were associated with reducing the risk of functional constipation compared to low-fiber and water intake, respectively (17). The prevalence of functional constipation was higher for infrequent physical activity (16.7%) (8.8–29.3) than those with frequent physical activity (9.1%) (5.5–14.6; *p* < 0.001) (17).

Due to many different lifestyle risk factors of constipation and their non-specific symptoms, it requires a careful analysis of the clinical history and a physical examination, as well as the exclusion of other anatomical disorders that also could alter intestinal function. To standardize the diagnostic criteria for CC, researchers from the non-profit organization, The Rome Foundation, elaborated on the Rome I, which contains four symptoms (straining to evacuate, lumpy or hard stool, and a sensation of incomplete evacuation, less than three evacuations per week), that must be present for 3 months (18). In Rome II, two additional symptoms (sensation of anorectal obstruction/blockage and manual maneuvers to facilitate defecation) were (18) included. In Rome III and IV, a new time criterion was added: the duration of the symptoms should have been initiated about 6 months before the diagnosis and must be present during the previous 3 months. In Rome IV, a new classification was added, opiate-induced constipation associated with chronic use of opioid medication (18).

Thus, using Rome IV criteria, the clinical physician can classify patients as having functional constipation (FC), irritable bowel syndrome with constipation (IBS-C), or opioid-induced constipation (OIC) (2). FC is a functional bowel disorder, in which symptoms such as difficult, infrequent, or incomplete defecation predominate. In IBS-C, abdominal pain is a predominant symptom, unlike FC. OIC differs from other types, due to its etiology, which develops secondary to the opioid effect on the GI tract and central nervous system (19).

Another widely used tool is the BSF, which is a validated 7-point scale (ranging from hard lumps to liquid consistency) to assess stool consistency. Moreover, a variety of questionnaires specific to the population with constipation had been developed such as the Cleveland Clinic Constipation Score (CCCS) to assess the severity of constipation in eight factors (frequency of bowel movements, difficulty, completeness, pain, time, assistance, failure, and history of constipation), with 0 being no constipation and 30 being severe constipation, and the Patient Assessment of Constipation Symptom (PAC-SYM) has become an important tool for measuring the severity of patient-reported symptoms of constipation in three categories: stool, rectal, and abdominal symptom (20, 21). Therefore, the choice of instrument to be used must be made according to the purpose of the investigation.

### Effect of prebiotic on CC

Initially, a prebiotic was described as a non-digestible food ingredient that beneficially affects the host by selectively stimulating the growth and/or activity of one or a limited number of bacteria in the colon, thus improving host health (22). According to this definition, a restricted number of compounds could be classified as prebiotics, such as short- and long-chain β-fructans (FOS and inulin), lactulose, and GOS. However, in the International Scientific Association for Probiotics and Prebiotics (ISAPP) consensus (2017), a new definition of prebiotic was proposed as a substrate that is selectively utilized by host microorganisms conferring a health benefit (23).

Few animal studies evaluating the effect of prebiotics on constipation were found (*n* = 6) (Supplementary Table 1). Among them, great diversity is observed, mainly between the prebiotic, such as lactulose, inulin, tagatose, sodium carboxymethyl starch, and different types of oligosaccharides (GOS, FOS, from lotus seed) and combined GOS + lactulose. Most animal studies (67%) evaluated other prebiotic dose groups (low, medium, and high doses) for 7 days (24–26) to 36 days of supplementation (27). The outcomes assessed in animal studies focused mainly on the number of stools, GI or colonic transit time/rate, and the water content of the stools.

Overall, prebiotics seem to have a beneficial effect on all constipation-related outcomes in constipated-induced animals. The most promising prebiotic dose on GI transit rate seems to be the medium (0.6–0.85 g/kg) (25, 26) and high dose groups (1.70–2.49 g/kg) (25, 26, 28). Regarding the defecation frequency/day, all doses evaluated show a beneficial effect; however, in the Liang et al.’s (25) study, only the low and medium doses of D-tagatose groups showed a significant increase in this outcome, with no difference in the high-dose group when compared to the constipated control group.

Only one study evaluated a combination of prebiotics in an animal model (27); in the Han et al.’s (27) study, galactooligosaccharides + lactulose increased the defecation frequency and reduced colonic transit time compared to the control group. However, more studies evaluating the effect of different types of prebiotics are needed to establish further conclusions on gut motility in constipation-induced animals.

Among human studies (*n* = 8), inulin (isolated or combine) was the most evaluated prebiotic (*n* = 4). Overall, the duration of prebiotic supplementation ranged from 14 (29) to 84 days (30). In the Glibowski et al. and Micka et al. (29, 31) studies, inulin supplementation increased the defecation frequency. However, in Micka et al.’s (31) study, no significant difference in stool consistency or constipated-associated symptoms was found, compared with the placebo (31). Inulin has a beneficial effect by modulating gut microbiota, increasing *Bifidobacterium* species, and lowering the *Bilophila* abundance rate. The decrease in these genera was associated with better QoL in healthy adults (32). When combined inulin with other agents, such as lactitol and aloe vera, no significant benefit for any evaluated outcome parameters was found (33).

Other prebiotic contents, such as lactulose alone, oligosaccharide, psyllium, and starch-entrapped microspheres, show no significant difference in constipated-related outcomes evaluated compared to the placebo group.

### Effect of probiotic on CC

Over the years, the definition of probiotics has changed; the most established definition by the scientific community is that probiotics are live microorganisms that, when administered in adequate amounts, confer a health benefit on the host (34). This definition was proposed by the International Scientific Association of Probiotics and Prebiotics (ISAPP) in 2014, which made a slight grammatical modification to the previously proposed by FAO/WHO in 2001 (35).

A great variety of studies conducted on humans and animals suggest the beneficial effects of probiotics on constipation-related outcomes (Supplementary Table 2). However, there are still conflicting data, possibly due to differing methodologies used as evidenced by systematic reviews of the use of probiotics in adults with constipation, which found high statistical heterogeneity among the studies (9, 11).

In animal studies, the most commonly used animal to induce constipation was mice, mainly Sprague-Dawley (*n* = 5), Kunming mice (*n* = 4), and BALB/c (*n* = 4), except for one study using zebrafish (36). The majority of animal studies evaluated a single probiotic strain (*n* = 13), mainly *Lactobacillus* (37–44) or *Bifidobacterium* genera (45–50). The duration of probiotic supplementation ranged from 4 (47) to 28 days (40, 41, 50). Overall, the most evaluated related-constipation outcome was GI transit. Probiotic supplementation seems to improve the GI transit/rate in animal models, as well as the number of stools, stool water content, and intestinal peristaltic movements.

Among the probiotic strains evaluated in animal studies, *L. plantarum* supplementation showed some controversial results. In studies by Gan et al. (37) and Kim et al. (51), there was no significant effect on CTT, whereas Eor et al. (52), Li et al. (38), and Zhao et al. (44) studies found a significant decrease in CTT/intestinal transit ratio. One of the hypotheses may be the low dose and short duration of supplementation (37) or its combined use with other probiotic strains (51).

In human studies, there were 19 studies evaluating single-strain probiotics (53–71). Of those, the most assessed strain was *Bifidobacterium lactis* (HN019, DN-173010, NCC2818, Bi-07, GCL2505) (*n* = 7) (53, 55–57, 64, 65, 71) and *Lactobacillus Casei Shirota* (*n* = 5) (54, 59, 61, 63, 69). Overall, only seven studies evaluated multistrain probiotics in humans (51, 72–77), with *Lactobacillus acidophilus* (*n* = 5) (51, 72, 73, 75, 76) and *Bifidobacterium lactis* (*n* = 4) (72, 73, 75, 77) species being the most frequent on multistrain probiotic content.

According to Supplementary Table 2, single-strain probiotic studies seem to have more effect on defecation frequency, stool consistency, and constipation-related symptoms, compared to multistrain probiotic studies. *L. casei Shirota* probiotics decreased several constipation symptoms such as pain, straining, and incomplete feeling during defecation (54, 69), abdominal discomfort (54, 69), and flatulence (59), as well as increased defecation frequency (59, 63, 66, 69) and stool consistency (54, 59, 63). *B. lactis* probiotic seems to have a beneficial effect mainly on defecation frequency (56, 57, 65, 66), while the results on stool consistency (55–57, 62, 66) and GI symptoms are still controversial (55, 57).

The dose of probiotics has also been a target of the investigation. Some human studies evaluated only high doses (≥ 10^10^ CFU) (*n* = 12), others evaluated low doses (< 10^10^ CFU) (*n* = 12), and a few investigated the effect of high *vs.* low dose on constipation (*n* = 2) (57, 64). Ibarra et al. and Waller et al. (57, 64) aimed to compare the effect of low *vs.* high doses of *B. lactis.* A beneficial improvement was seen in high- and low-dose groups compared to placebo, in the abdominal pain, constipation, flatulence, and defecation frequency outcomes (64). In Ibarra et al.’s study, both probiotic dose groups had a beneficial effect on defecation frequency in those participants with ≤ 3 times/week in baseline; however, a decrease in the degree of straining symptoms was only observed in the high-dose group (57). Although the effect of a high dose of probiotics was superior in only one symptom, a recent systematic review found that there was no statistical difference between high and low doses. Therefore, high- and low-dose probiotic supplementation seems to have a positive effect on constipation-related symptoms, and a recent systematic review found no statistical difference between the high- and low-dose groups on defecation frequency, colonic transit, and stool consistency (9).

With regards to the duration of supplementation, most human studies evaluated a longer duration (≥ 28 days) (*n* = 19), rather than a shorter duration (< 28 days) (*n* = 9). No included study aimed to evaluate the different duration of probiotic consumption on constipation-related outcomes. A subgroup analysis of a meta-analysis evaluating the effects of *B. lactis* probiotic on GI symptoms showed that the shorter duration group had a superior effect compared to the longer duration group on the defecation frequency outcome (9); however, the best optimal duration time may vary from probiotic strain. Moreover, the effects of probiotics varied greatly between studies, due to different doses and duration of supplementation, but also due to different strains administrated, since probiotic is known to be strain-dependent (13).

The strain(s), dose, and duration of intake are well-known determining aspects to be considered for the probiotics to have their expected effects. However other characteristics of the usage of probiotics could also influence their effects and must be considered in future studies, such as the time of day of the probiotic intake. The time of day of probiotic intake varied widely between studies, such as 1 h after a meal (56), 30 min after the last meal (72, 73), 30 min after breakfast and dinner (76), during meals (64, 71), before/during meals (62, 75), between meals (78), after lunch (54, 69), without setting time for ingesting (53), or not reported (*n* = 15) (51, 55, 57–61, 63, 65–67, 70, 74, 77, 79). Previously, a study evaluating the time of probiotic administration showed that, when given 30 min after the meal, probiotics had a lower rate of survival when given before or during meals (80). Possibly due to the more hostile gastrointestinal environment during digestion (i.e., acidity, bile, and enzymes), which can reduce the effect of the probiotic, whereas, before the meal, these compounds have not yet been released. During the meal, the presence of food could protect the delivery of the probiotic to the intestine. However, to date, the optimal time to consume probiotics remains unknown.

Another further aspect to be considered in the upcoming randomized control trial (RCT) is how probiotics should be consumed (mixed with another food/beverage or alone). In several studies, probiotics were delivered on a food/beverage matrix, such as with milk (*n* = 1) or non-specific dairy product (*n* = 2) or yogurt (*n* = 1) or fermented milk (*n* = 5), cheese, beverage (*n* = 1), and artichokes (*n* = 1). Studies in which probiotics were delivered in capsules/sachet/tablets were dissolved in different contents such as water (*n* = 2), yogurt (*n* = 1), non-specific dairy product (*n* = 1), or not reported (*n* = 11). The difference in the probiotic matrix may be a potential cause for the wide variation in the observed probiotic effect. Although recent studies found no significant difference between yogurt and capsule matrix of *B. animalis* subsp. *lactis* BB-12 and *L. acidophilus* LA-5 (81) or *B. animalis* subsp. *lactis* BB-12 isolated (82), the use of cheese as a probiotic matrix was shown to be less effective in the adhesion of the probiotic strain to the GI tract (83). Thus, further RCTs are needed to assess whether there is a difference between probiotic matrices and whether there is a difference between the content where the probiotic capsules or sachets will be dissolved for consumption.

### Effect of synbiotic on CC

Synbiotics are the association of probiotics and prebiotics, defined as “a mixture comprising live microorganisms and substrate(s) selectively utilized by host microorganisms that confers a health benefit on the host” (84).

Compared to probiotics, studies evaluating the effect of synbiotics on constipation are scarcer both in human (*n* = 10) and animal models (*n* = 2). The most prebiotics used in synbiotic products were inulin (*n* = 4) (85–88), followed by FOS (*n* = 2) (89, 90) and psyllium (*n* = 2) (85, 91); only two studies of the synbiotic products contained a single probiotic strain, *B. coagulant* (92) or *B. animals* (88).

In addition to the lower number of studies, synbiotic supplementation has demonstrated some beneficial effects on constipation-associated outcomes (Supplementary Table 3). In humans, synbiotics may reduce CTT (87, 91, 93, 94), increase defecation frequency (88–90, 92, 93), and improve stool consistency (88–90, 92, 93), but seems to have a controversial effect on constipation-related symptoms (bloating, abdominal pain, and discomfort), with no significant effect on the PAC-SYM score (85, 86, 89, 93).

Therefore, more RCTs assessing the effect of synbiotics on constipation outcomes are still needed due to their controversial effect on constipation-related symptoms to better understand their effects on this condition and for future constipation treatment protocols that can be established.

### Possible mechanisms of action of prebiotic, probiotic, and synbiotic on CC

**FIGURE 1:**
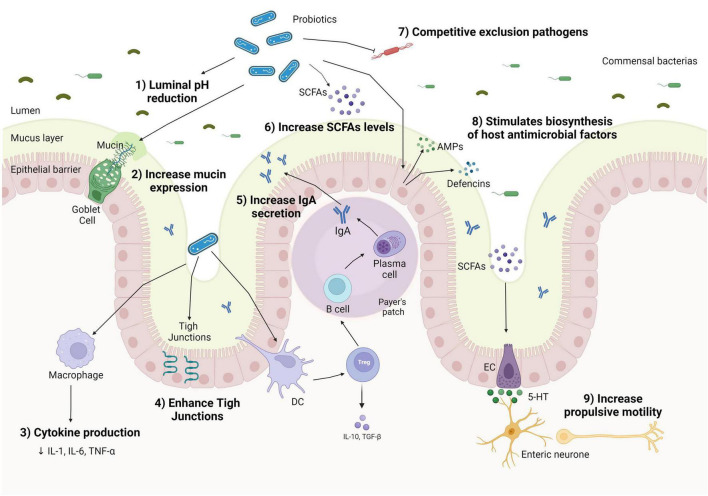
Mechanisms of action of probiotics on the human gut in chronic constipation. Probiotics can decrease luminal pH (1); increase mucin production by goblet cells (2); shift macrophage cytokines production, lowering pro-inflammatory cytokines production (3); enhance tight junction protein expression, improve gut barrier function (4); increase Treg expression and stimulating IgA to enhance immunoglobulin A (IgA) secretion in Peyer’s patch (5); stimulate the proliferation of short-chain fatty acid (SCFA) bacterial producers (6); inhibit the colonization of pathogenic bacteria by competing for nutrients and location (7); enhance the secretion of antimicrobial peptides (AMPs), such as defensins and cathelicidins by epithelial cells *via* activation of the innate response, helping to eliminate commensals or pathogens that penetrate the mucus layer (8); and increase colonic motility through the conversion of tryptophan hydroxylase 1–5-hydroxytryptophan (5-HTP), which are converted to 5-hydroxytryptophan (5-HT) and released by enterochromaffin cells (ECs) (9).

Several similar mechanisms have been proposed concerning the action of pre-, pro-, and synbiotics on CC. In the case of prebiotics, they can stimulate the proliferation of commensal bacteria in the colon and the production of local metabolites, due to the undergone fermentation by commensal bacteria, such as *Lactobacillus* and *Bifidobacteria* in the gut (95). The fermentation of prebiotics can induce the production of SCFAs, such as butyrate. The increase in SCFAs by prebiotic supplementation might inhibit pathogenic growth by lowering the pH of the small intestine and can also change gut motility by stimulating the contraction of colonic smooth muscles, ameliorating the constipation symptoms (95). However, these effects of prebiotics on SCFAs concentrations are still under discussion, because of the selective effect of different prebiotic materials and the rapid absorption of SCFAs by the epithelium and other gut bacteria, which can limit the assessment of SCFAs level from fecal samples. The supplementation of different prebiotics (e.g., insulin, lactitol, and aloe vera gel or GOS) did not change the fecal SCFA concentrations in healthy or constipated adults after the treatment (96, 97). However, an increase in butyrate producer bacterium was found, such as *Roseburia hominis*, a major butyrate producer (33). In Liu et al.’s (98) study, a significant decrease was observed in butyrate-producing bacteria, after 14 days of a high dose of FOS and GOS supplementation in healthy adults, possibly by the excessive increase in lactic acid promoted by *Bifidobacterium* proliferation, and also hindering the growth of butyrate-producing bacteria and SCFA production. Similarly, probiotics and synbiotics can modulate the gut microbiota by SCFA production, even more widely than isolated prebiotics (Figure 1). SCFAs can act as antibacterial substances, inhibiting the growth of pathogenic bacteria by diffusion across the bacterial membrane and decreasing their cytoplasmic pH *via* the accumulation of organic acids (99). This antimicrobial activity has been found by Liu et al. (100) in which *L. plantarum* ZS2058 inhibited the growth of *Salmonella* mediated by increasing propionic acid levels in mice.

The production of SCFAs is also related to anti-inflammatory actions in the intestinal mucosa by inhibiting histone deacetylase (HDAC) activity, promoting histone deacetylation, affecting cell regulation and proliferation and inflammatory response, and blocking the Toll-like pro-inflammatory receptor (TLR) in human dendritic cells (DCs) (101). Specific probiotic strains of *Bifidobacterium* and *Lactobacillus* can activate intestinal DCs, stimulating the expression of T cell (Treg) and IL-10 release and inducing a switch of macrophage phenotype pro-inflammatory to anti-inflammatory (M2) (102). The change in the profile of macrophages phenotype contributes to the improvement of bowel movements since the suppression of pro-inflammatory cytokines acts by preserving the signaling of the ENS and smooth muscle, resulting in the regulation of GI motility (103, 104).

Short-chain fatty acids can also stimulate the secretion of substances such as peptide YY (PYY) and glucagon-like peptide-1 (GLP-1) on enteroendocrine cells (105), which promote increased bowel movements and colonic transit. It can regulate motility by activating the cell body (soma) of myenteric intrinsic primary afferent neurons (IPANs) derived from blood (106). They are also capable to promote the expression of the tryptophan hydroxylase-1 (TPH1) enzyme in the colon (107). TPH1 enzyme can stimulate the production of tryptophan 5-hydroxylase 1 (5-HT) on intestinal epithelial cells and also the release of 5-HT by mast cells, which stimulate propulsive contractions in the ileum, favoring intestinal motility and decreasing CTT (107). However, a possible adverse effect of a high concentration of SCFA as an association with obesity has been discussed (108).

Another mechanism of probiotics is the stimulation of mucin secretion by the increase in mucin (MUC) gene expression and the activity of goblet cells (109). The mucus layer can act as a lubricant, facilitating stool passage (110) and serving as a protective antimicrobial substance. Probiotics can also improve the gut barrier function by upregulating the expression of tight junctions’ proteins and, consequently, inhibiting the adherence of pathogen bacteria, and their metabolites on the intestinal epithelial barrier.

Moreover, when there is a pathogen recognition receptor (PRR), probiotics can stimulate the epithelial cells and Paneth cells to produce antimicrobial peptides (AMPs), such as defensins and cathelicidins, which present a high antimicrobial activity, and promote competitive exclusion (111), as demonstrated by *L. acidophilus* and *L. fermentum* supplementation on inhibiting pathogenic strains (112).

Probiotics may also have a potential impact on inflammatory regulation and could be a promising supplementation on inflammation associated with constipation since inflammatory response had been reported in some individuals with constipation (113). In addition to the mechanisms previously cited, probiotics also increase the secretory of immunoglobulin A (IgA) through dendritic cell stimulation, which controls bacterial translocation and neutralizes bacterial toxins at the intestinal mucosal surface (114).

With regard to synbiotics, they confer the prebiotic and probiotic benefits and their synergistic effects, potentially ensuring the increase in SCFAs, improving tight junctions and mucin production, lowering the intestinal pH, and balancing gut microbiota composition (115). However, synbiotics are the least investigated substances regarding health effects, compared to prebiotics and probiotics, and their mechanisms still need to be better understood.

### Outstanding factors to be considered for probiotic use on constipation

#### Probiotic supplementation and polymorphisms on constipation

As previously mentioned, serotonin (5-HT) is an important gastrointestinal neurotransmitter, which regulates peristalsis in the gastrointestinal tract. The concentration and duration of 5-HT are mainly determined by the serotonin-selective reuptake transporter (SERT), *via* the mediation of extracellular reuptake and recycling of 5-HT (116).

The serotonin-selective reuptake transporter polymorphism results in insertion (L) and deletion (S) alleles. In functional studies using a transfected cell line, homozygous deletion (S/S) and heterozygous SERT (L/S) genotypes were associated with lower transcriptional activity compared to that of the homozygous insertion genotype (L/L), leading to a reduction in 5-HT reuptake and consequently increasing 5-HT levels (116).

Studies investigating the association between SERT gene polymorphism and constipation focus on patients with cancer (117) or irritable bowel syndrome (IBS) (118, 119). In Li et al.’s (117) study, patients with polymorphism in S/S genotype SERT have a higher risk for constipation. In Cengiz Pata et al. and Zhu et al. studies (118, 119), it was observed a significant association between SERT polymorphism with predominant constipation IBS (IBS-C). The polymorphisms in SERT genes could lead to downregulation in the 5-HT receptors over time, decreasing the serotonergic effect, which leads to constipation (119).

In intestinal epithelial cells and mice intestinal tissues, *L. acidophilus* and *B. longum* (120), as well as *L. rhamnosus* GG supernatant (LGG-s) (121) administration increased SERT expression. However, to the best of our knowledge until now, only one RCT investigated the polymorphism in patients with functional constipation (*n* = 56) (68). Riezzo et al. (68) observed that those patients with the S allele of the 5-HTT gene-linked polymorphic region (5-HTTLPR) in the SERT gene reduced 5-HT concentration after 105 days of *Lactobacillus reuteri* (LR) DSM 17938 supplementation. Thus, this study suggests that probiotic use may improve the expression of SERT in the intestinal epithelium and increase 5-HT reuptake, indicating that patients with the S allele of the 5-HTTLPR may benefit from LR DSM 17938 supplementation (68).

Evidence on the effect of polymorphisms on constipation, as well as the effect of probiotics on the management of constipation *via* 5-HT, although promising, is still scarce. Thus, further studies are needed to elucidate the physiological importance of gene polymorphisms in the pathogenesis and treatment of constipation.

#### Probiotic supplementation on clinical practices

To date, several animal and human studies are suggesting a probiotic effect on constipation-related outcomes. Recent meta-analyses suggest promising effects of the *B. lactis* strain on increasing defecation frequency, CTT, and stool consistency; however, a high heterogeneity and bias across studies were observed, and thus, caution is still needed in interpreting these findings (9, 39).

This heterogeneity of the protocol used across studies also makes it difficult to define a better dose and treatment time. Even so, most studies did not consider the main influencing factors of constipation in their final analysis, such as age, sex, changes in food consumption, physical activity, and alcohol intake during the intervention. These factors should be evaluated and considered in future studies to determine the actual effect of probiotics on constipation, due to their impact on gut microbiota composition and motility (122). Another relevant concern is the inclusion of different types of constipation in the RCT, due to its singular characteristics. Among the human studies assessing the effect of probiotics (*n* = 26), six did not report the participant’s type of constipation, and the other 20 included only participants with functional constipation. However, all of those do not specify the subtype of functional constipation. Including participants with different types and subtypes of constipation may mask the actual effects of the probiotics and increase the heterogeneity of the results found among studies. Therefore, future studies should evaluate the different types and subtypes of constipation and consider them in their final analysis.

Even though it is still difficult to establish with current evidence which probiotic strain is the most clinically effective, clinicians have begun to incorporate probiotics as a non-pharmacological therapy option for constipation. An online survey of 1,066 healthcare professionals from 30 countries (123) found that 79% of professionals evaluated had already advised their patients to use probiotics, and, regarding the recommendation of probiotics for patients with constipation, another survey found from 1,830 primary care health professionals evaluated, 18% recommend probiotics (124).

Among the probiotic strains, *B. lactis* showed a beneficial effect mainly on defecation frequency outcome, while *L. casei Shirota* probiotics improved several constipation symptoms and stool consistency, along with increased defecation frequency. Although we do not have yet enough evidence to establish the best probiotic strain for constipation-associated outcomes, it can be started with a minimum daily dose of 10^9^ CFU, accompanied by periodic follow-up to determine the most recommended dose according to the individual response. In addition, it is not enough to use probiotics and maintain an inappropriate lifestyle and diet, given that these factors also modulate the gut microbiota and can contribute to constipation and its severity (17). Thus, the prescription of probiotics should be associated with dietary intervention and physical activity, given the influence of these factors on constipation.

Although the current study highlighted some relevant outstanding features of probiotics, prebiotics, and synbiotics on CC, they are some limitations. Despite PubMed being a major health clinical articles database, using a single database could limit the number of potentially included studies. Another relevant limitation is the change in the prebiotic definition that could lead to missing potential studies on the data search.

## Conclusion

The supplementation of prebiotics, probiotics, and synbiotics may serve as useful alternatives to improve constipation-related outcomes. Among prebiotics, inulin showed to be the most promising type to increase defecation frequency. In terms of synbiotics, despite their effect on the reduction in CTT, increase in defecation frequency, and improvement in stool consistency, there is a controversial effect on constipation-related symptoms; therefore, more studies are needed to better understand their effects and mechanism. To date, probiotics have been the most studied dietary supplementation for the treatment of constipation. Although the best probiotic strain for constipation is still debated, *B. lactis* demonstrated a beneficial effect on defecation frequency, while *L. casei Shirota* improved several constipation symptoms and stool consistency, along with increased defecation frequency, suggesting that the probiotic effect on constipation symptoms may be strain-dependent. Moreover, there are still some features of the use of probiotics in constipation-related outcomes that have not yet been answered such as the best dose (high or low dose), duration (shorter or longer), time of consumption (before, during, or after meals), probiotic matrices, as well as their effect and mechanisms on the regulation of inflammation in patients with constipation, on polymorphisms associated with constipation, and on the management of constipation *via* 5-HT. Therefore, further high-quality RCTs evaluating different protocols are needed to confer secure conclusions regarding the effectiveness of probiotics and the best usage protocol.

## Author contributions

PB developed the idea and manuscript structure for the review and commented critically on the manuscript drafts. MA undertook the literature search and wrote the manuscript. Both authors contributed to the article and approved the submitted version.
