# Supplementary material for: Probiotics, prebiotics, and synbiotics in chronic constipation: Outstanding aspects to be considered for the current evidence
Source: Front Nutr. 2022 Dec 8;9:935830. doi: 10.3389/fnut.2022.935830 (PMC9773270; doi:10.3389/fnut.2022.935830)
Supplement: Supplementary file 1 [file Data_Sheet_1.docx]

Supplementary Material

**Supplementary Table 1.** Studies evaluating the effect of prebiotics on the gastrointestinal tract in animal models and human studies.

| **Reference** | **Study Design†** | **Sample (n; sex)** | **Participants Characteristics**  **(age; health condition)** | **Control** | **Intervention** | **Period of supplementation** | **Main**  **Results**  **(compared to control group)** |
| --- | --- | --- | --- | --- | --- | --- | --- |
| **ANIMAL MODELS** | | | | | | | |
| Han 2016 (1) | Experimental trial | 32; N/E | mice (Sprague-Dawley);  25 days old;  loperamide-induced constipation | non-constipated control group= standard diet, n=8;  constipated control group= loperamide, n=8 | Group low dose: Dual-Oligo (10%); n= 8  Group high-dose: Dual-Oligo (15%) n= 8  All: 1x/d; dissolved in water | 36 days | ↑ CTT  ↑Defecation frequency |
| Lan 2020 (2) | Experimental trial | 24 males; | Mice;  6 weeks old;  diphenoxy -induced constipation | non-constipated control group= standard diet, n=6;  constipated control group= diphenoxy-  late, n=6; | Inulin group= 20g/kg inulin, n= 6,  Isomalto-oligosaccharide group= 20g/kg  Isomalto-oligosaccharide, n= 6 | 7 days | ↑ Fecal number  ↑ Water content of fecal pallets (in both intervention groups) |
| Liang 2019 (3) | Experimental trial | 50 males | mice (Kunming);  N/E weeks old;  loperamide-induced constipation | non-constipated control group= standard diet, n=10;  constipated control group= loperamide, n=10; | Group low dose: of 0.43 g/kg D-tagatose, n=10  Group medium dose: 0.85 g/kg D-tagatose, n=10  Group high dose: 1.70 g/kg D-tagatose, n=10 | 7 days | ↑ GI transit rate (in medium and high dose groups)  ↑ Fecal number (in low and medium dose groups) |
| Lu 2021 (4) | Experimental trial | 50; N/E | mice (Kunming);  N/E weeks old;  loperamide-induced constipation | non-constipated control group= standard diet, n=10;  constipated control group= loperamide, n=10; | Group low dose= 0.3g/kg Sodium carboxymethyl starch (CMS-Na), n= 10;  Group medium dose= 0.6g/kg CMS-Na, n= 10;  Group high dose= 1.2/kg CMS-Na, n= 10;  Mosapride group= 1.2mg/kg mosapride.n= 10; | 7 days | ↑ Fecal number (in three groups)  ↑ Water content of fecal pallets (in three groups)  ↓ CTT (in medium and higher dose groups) |
| Su 2019 (5) | Experimental trial | 98 (50:50 males:females); | mice (BALB/c);  4 weeks old;  loperamide-induced constipation | non-constipated control group= standard diet, n=14;  constipated control group= loperamide, n=14; | Group low dose= 0.42 g/kg lotus seed oligosaccharides, n= 14;  Group medium dose= 0.83 g/kg lotus seed oligosaccharides, n= 14;  Group high dose= 2.49 g/kg lotus seed oligosaccharides, n= 14;  Group ORG= 0.21 g/kg lotus seed oligosaccharides + diet with 5% resistant starch, n=14;  Group FG= 0.83 g/kg of FOS, n= 14 | 15 days | ↑ Ink propulsion rates (in high dose and ORG groups)  ↓ Defecation time (in high dose, medium dose and ORG and FG groups) |
| Zhang 2021 (6) | Experimental trial | 48 males | mice (BALB/c);  6 weeks old;  loperamide-induced constipation | non-constipated control group= standard diet, n=12;  constipated control group= loperamide, n=12; | Group LAC: non-constipated mice treated with 2.5 g/kg/d Lactulose, n=12;  Group LOP+LAC: loperamide-induced constipation + 2.5 g/kg/d Lactulose, n=12; | 14 days | ↑ GI transit rate  ↑ Number of feces |
| **HUMANS STUDIES** | | | | | | | |
| Bouhnik 2004 (7) | RCT | 65; 56 females | All: 57 ± 18 years;  With chronic idiopathic constipation by Rome I diagnostic criteria | Polyethylene glycol (10g); n= 32 | Lactulose (10g); sachet; | 28 days | No significant differences on abdominal pain, flatus in excess, bloating, stool consistency. |
| Chu 2019 (8) | RCT | 40; 30 females | Control: 24 (21-27) years; Prebiotic: 25 (22-51) years;  < 3 bowel movements/week, with sensation of incomplete evacuation (> 25%); and straining with defecation (> 25%), during 3-6 months | Maltodextrin; n= 20 | Inulin (61.5%), lactitol (34.6%), and aloe vera gel (3.9%) | 28 days | No significant differences on gastrointestinal symptoms, stool consistency; defecation frequency. |
| Glibowski 2020 (9) | RCT not-blind | 20; N/E | N/E years; with chronic constipation self-reported | Juice; n= 10 | Inulin (12g/300mL); n=10 | 14 days | ↑ Defecation Frequency.  ↑ Ease of defecation.  No significant differences on flatulence or bloating |
| Micka 2016 (10) | RCT | 44; 33 females | All: 46.9 ± 13.2 years  constipation defined as 2–3 stools per week for at least 6 months | Maltodextrin; n= N/E | Inulin (12g); 3x/d; n= N/E; sachet; with main meals (breakfast, lunch, dinner); dissolved in drinks | 28 days | ↑ Defecation Frequency.  No significant differences on stool consistency, straining, feeling of incomplete emptying and bloating/distension. |
| Muller 2020 (11) | RCT | 48; 12 females | Prebiotic: 36.1 ± 12.9 years  Control: 35.7 ± 11.0 years | Maltodextrin; n=24 | Arabinoxylan-Oligosaccharide (15g); n=24; sachet; 3x/d; their regular meals; dissolved in water | 84 days | No significant differences on stool consistency and colonic transit time. |
| Rasmussen 2017 (12) | RCT | 43; 30 females | All: 42.6 ±11.9 years;  < 3 bowel movements/week, and hard stool requiring straining, or sense of  incomplete defecation) | non-control group | Starch-entrapped microspheres (SM) (9g); n= 15;  Starch-entrapped microspheres (SM) (12g); n=15;  Psyllium (12g); n= 13  All: Capsules 2x/d | 21 days | ↑ Defecation Frequency*  No significant differences on stool consistency. |
| Vandeputte 2016 (13) | RCT cross-over | 44; 33 females | All: 46.9 ± 13.2 years;  mild constipation | Maltodextrin; n= NE | Inulin (12g); 1x/d | 28 days | N/E |

↑, Increased or Improve; ↓, Decreased; †Double-blind placebo-control, unless otherwise stated; GI: Gastrointestinal; RCT : Randomized Controlled Trial. CTT: Colonic Transit Time. N/E: Not reported. * Significant difference only within-group. Dual-Oligo content: galactooligosaccharides and lactulose; FOS: Fruto-oligossacharideo.

**Supplementary Table 2.** Studies evaluating the effect of probiotics on the gastrointestinal tract in animal models and human studies.

| **Reference** | **Study Design†** | **Sample (n; sex)** | **Participants Characteristics**  **(age; health condition)** | **Control** | **Intervention** | **Period of supplementation** | **Main**  **results** |
| --- | --- | --- | --- | --- | --- | --- | --- |
| **ANIMAL MODELS** | | | | | | | |
| Deng, 2018 (14) | Experimental trial | 21; all males | mice (Sprague Dawley); 8 weeks old;  loperamide-induced constipation | non-constipated group= phosphate buffered saline, n= 7  constipated induced group= phosphate buffered saline, n=7 | Probiotic group: *Bifidobacterium breve* DM8310, *Lactobacillus acidophilus* DM8302, and *Lactobacillus casei* DM8121; 1×10^9^ CFU/kg bacterial mixture suspended in 2 ml PBS; 1x/d, n= 7 | 7 days | ↑ Small intestine transit rate (SIT) compared to the constipated group. |
| Eor, 2018 (15) | Experimental trial | 56; all females | mice (Sprague Dawley);  8 weeks old; loperamide-induced constipation | non-constipated group= phosphate buffered saline, n=14;  constipated group= phosphate buffered saline, n=14;  constipated group +chocolate = phosphate buffered saline + chocolate n=14; | Probiotic group: Chocolate + *S. thermophilus* MG510 and *Lactobacillus plantarum* LRCC5193; 2 x 10^8^ CFU; 1x/d, n= 14 | 14 days | ↓ CTT (probiotic group) compared to constipated group. |
| Gan, 2020 (16) | Experimental trial | 50; all males | mice (Kunming);  6 weeks old; montmorillonite-induced constipation | Non-constipated group= standard diet, n= 10  Constipated group= standard diet + montmorillonite, n= 10  geniposide group: geniposide + standard diet; n= 10 | Probiotic: *Lactobacillus plantarum* KSFY06, 0.5 × 10^7^ CFU/kg; 1x/d; n=10  Probiotic + geniposide group: *Lactobacillus plantarum* KSFY06 1 × 10^7^ CFU/kg + geniposide 50 mg/kg; n=10 | 9 days | No significant difference on CTT. |
| Hayeeawaema, 2020 (17) | Experimental trial | N/E | mice (ICR/Mlac); 6 weeks old; loperamide-induced constipation | non-constipated group: standard diet; n= N/E  constipated group: standard diet + loperamide; n= N/E | Probiotic group: *Bifidobacterium animalis subsp. lactis* Bb-12; 1x10^9^ CFU; 1x/d; n= N/E  Prebiotic group: Lactulose 500mg/kg 1x/d; n= N/E  KGM group: KGM 100mg/kg; 1x/d; n= N/E  KOG_low-dose_ group: KOG 100mg/kg; 1x/d; n= N/E  KOG_midium-dose_ group: 500mg/kg; 1x/d; n= N/E  KOG_high-dose_ group: 1000mg/kg; 1x/d; n= N/E | 14 days | ↓ CTT (probiotic, prebiotic, KGM and the dose of KOG groups) compared to the constipated group. |
| Kim, 2021 (18) | Experimental trial | 24; all males | mice (Sprague-Dawley); 6 weeks old; loperamide-induced constipation | non-constipated group= standard diet, n= 6  constipated induced group= standard diet,  n= 6 | Probiotic: *Lactobacillus plantarum, L. acidophilus, Bifidobacterium bifidum, B. lactis,* and *Streptococcus thermophilus*; 31 mg/kg; 1x/d; n= 6  Synbiotic: *Lactobacillus plantarum, L. acidophilus, Bifidobacterium bifidum, B. lactis,* and *Streptococcus thermophilus* + lactitol; 31 mg and 120 mg/kg, respectively; 1x/d; n= 6 | 21 days | No significant difference on CTT. |
| Lee 2018 (19) | Experimental trial | 60; all females | mice (Sprague-Dawley) sixty 8 weeks old; loperamide-induced constipation | non-constipated group= standard diet; n= 12  constipated induced group= standard diet; n=12  constipated induced group treated with chocolate= standard diet + chocolate, n= 12 | Probiotic BB12= chocolate with *Bifidobacterium animalis* subsp. *lactis* BB-12 + chocolate powder (2.5 × 10^10^ CFU); 1x/d; n=12  Probiotic LYC group= chocolate with 2.5 × 10^10^ CFU mL of *S. thermophilus* MG510 and *Lactobacillus plantarum* LRCC5193; 1x/d; n= 12 | 15 days | No significant difference in intestinal transit ratio. |
| Li 2015 (20) | Experimental trial | 50; all males | mice (Kunming);  loperamide-induced constipation | non-constipated group= standard diet; n= 10  constipated induced group= standard diet; n= 10 | Probiotic _low-dose_= *Lactobacillus plantarum* NCU116 (1x107 CFU); n= 10  Probiotic _medium-dose_= *Lactobacillus plantarum* NCU116 (1x108 CFU); n= 10  Probiotic _high-dose_= *Lactobacillus plantarum* NCU116 (1x109 CFU); n= 10 | 15 days | ↑ intestinal transit ratio in all three probiotic groups  No significant difference on fecal pallet number in all probiotic groups. |
| Lu 2019 (21) | Experimental trial | 220; N/E | zebrafish; loperamide-induced constipation | non-constipated group= standard diet; n= 20  constipated induced group= standard diet; n= 20 | Probiotic X11= *Lactobacillus paracasei* (X11); 10^8^ CFU/mL; n=20  Probiotic BB-12= *Bifidobacterium lactis*; 10^8^ CFU/m; n= 20  Probiotic K11= *Lactobacillus. casei* K11; 10^8^ CFU/m; n= 20  Probiotic LGG= *Lactobacillus rhamnosus* GG; 10^8^ CFU/m; n= 20  Probiotic KV9= *Bifidobacterium lactis* KV9; 10^8^ CFU/m; n= 20  Probiotic MS= *L. paracasei MS*; 10^8^ CFU/m; n= 20  Probiotic YLD= *Lactobacillus casei* YLD; 10^8^ CFU/m; n= 20  Probiotic YRT3115= *Bifidobacterium bifidum* YRT3115; 10^8^ CFU/m; n= 20  Drug group= domperidone; n=20 | N/E | ↑ Intestinal peristalsis (in all groups, and probiotic X11 and BB-12 groups had a better effect) |
| Makizaki, 2021 (22) | Experimental trial | 48; all males | mice (male Sprague Dawley);  7 weeks old; loperamide-induced constipation | non-constipated group= standard diet, n= 16  constipated induced group= standard diet; n= 16 | Probiotic= *Bifidobacterium bifidum* G9-1 (1x10^10^ CFU); 3x/d; n= 16 | 4 days | ↑ Fecal number; ↓ Fecal hardness.  ↑ Intestinal peristaltic |
| Tan 2021 (23) | Experimental trial | 50; all females | mice (ICR);  6 weeks old; loperamide-induced constipation | non-constipated group= standard diet; n= 10  constipated induced group= standard diet; n= 10 | Probiotic _low-dose_: *Lactococcus lactis* subsp. *lactis* (1×10^8^ CFU/kg) n= 10  Probiotic _High-dose_: *Lactococcus lactis* subsp. *lactis* (1×10^9^ CFU/kg); n= 10  Lactulose group= 3g/kg lactulose; n= 10 | 28 days | ↑ Fecal number (both probiotic groups); |
| Wang, 2017 (24) | Experimental trial | 88; all males | mice (BALB/c);  7 weeks old; loperamide-induced constipation | non-constipated group= standard diet; n= 8  constipated induced group= standard diet; n= 8 | Probiotic CCFM 626 _low-dose_: *Bifidobacterium adolescentis* CCFM 626*;* 1 × 10^8^ CFU; n= 8  Probiotic CCFM 626 _high-dose_: *Bifidobacterium adolescentis* CCFM 626; 1 × 10^10^ CFU; n= 8  Probiotic CCFM 667 _low-dose_: *Bifidobacterium adolescentis* CCFM 667*;* 1 × 10^8^ CFU; n= 8  Probiotic CCFM 667 _high-dose_: *Bifidobacterium adolescentis* CCFM 667; 1 × 10^10^ CFU; n= 8  Probiotic CCFM 669 _low-dose_: *Bifidobacterium adolescentis* CCFM 669*;* 1 × 10^8^ CFU; n= 8  Probiotic CCFM 669 _high-dose_: *Bifidobacterium adolescentis* CCFM 669; 1 × 10^10^ CFU; n= 8  Drug group= phenolphthalein 70 mg/kg; n= 8  Probiotic ST-III _low-dose_: *L. plantarum* ST-III; 1 x 10^8^ CFU; n= 8  Probiotic ST-III _high-dose_: *L. plantarum* ST-III; 1 x 10^10^ CFU; n= 8 | 17 days | ↑ GI transit rate (except for both low and high dose Probiotic CCFM 626 groups) |
| Wang 2019 (25) | Experimental trial | 32; all males | mice (BALB/c);  8 weeks old; loperamide-induced constipation | non-constipated group= standard diet; n=8  constipated induced group= standard diet; n=8 | CMB1: *Bifidobacterium longum* CCFM 643 *Bifidobacterium breve* CCFM 670 *Bifidobacterium bifidum* CCFM 16 *Bifidobacterium adolescentis* CCFM 669 *Bifidobacterium animalis* CCFM 6254; (4 ×10^10^ CFU/mL); strains with adherence property; n=8  CMB2: *Bifidobacterium longum* CCFM 642 *Bifidobacterium breve* CCFM 622 *Bifidobacterium bifidum* CCFM 641 *Bifidobacterium adolescentis* CCFM 626 *Bifidobacterium animalis* CCFM 624; (4 ×10^10^ CFU/mL); strains without adherence property; n=8 | 17 days | ↑ Fecal number and small intestinal transit rates (in CMB1) |
| Wang, 2020 (26) | Experimental trial | 40; all males | mice (BALB/c);  6-8 weeks old; loperamide-induced constipation | non-constipated group= standard diet; n=10  constipated induced group= standard diet; n=10 | Probiotic _low-dose_*= Bifidobacterium animalis* subsp. *lactis* MN-Gup; (2x10^9^ CFU/kg), 1x/d; n=10  Probiotic _high-dose_*=Bifidobacterium animalis* subsp. *lactis* MN-Gup (2x10^10^ CFU/kg), 1x/d; n=10 | 28 days | ↑ Small intestinal transit rates (in both probiotic groups)  ↑ Fecal water content; fecal number (in high-dose group) |
| Wang, 2020 (27) | Experimental trial | 42; all males | mice (BALB/c);  6 weeks old; loperamide-induced constipation | non-constipated group= standard diet; n= N/E  constipated induced group= standard diet; n= N/E | Probiotic CCFM 1068= *Lactobacillus rhamnosus* CCFM 1068 (5 ×10^9^ CFU/mL); n= N/E  Probiotic FFJND15-L2= *Lactobacillus rhamnosus* FFJND15-L2 (5 ×10^9^ CFU/mL); n= N/E  Probiotic FHeNJZ7-1= *Lactobacillus rhamnosus* FHeNJZ7-1 (5 ×10^9^ CFU/mL); n= N/E  Probiotic FTJDJ11-1= *Lactobacillus rhamnosus* FTJDJ11-1 (5 ×10^9^ CFU/mL); n= N/E  Probiotic FZJHZ11-7= *Lactobacillus rhamnosus* FZJHZ11-7 (5 ×10^9^ CFU/mL); n= N/E | 28 days | ↓ GI transit time (in all probiotic groups)  ↑ Fecal water content (in CCFM 1068 group) |
| Zhang 2018 (28) | Experimental trial | 50; all females | mice (Kunming); 6 weeks old; loperamide-induced constipation | non-constipated group= standard diet; n= 10  constipated induced group= standard diet; n= 10 | Probiotic LB= *Lactobacillus bulgaricus* (LB); 1x10^9^ CFU/kg; n= 10  Probiotic CQPC03 _low-dose=_ *Lactobacillus fermentum* CQPC03 (1x10^8^ CFU/kg); n= 10  Probiotic CQPC03 _high-dose=_ *Lactobacillus fermentum* CQPC03 (1x10^9^ CFU/kg) ; n= 10 | 17 days | ↑ Intestinal peristaltic and Fecal water content on 8-10 days (in all probiotic groups) |
| Zhao, 2015 (29) | Experimental trial | 120; all females | mice (ICR); 7 weeks old; loperamide-induced constipation | non-constipated group= standard diet; n= 24  constipated induced group= standard diet; n= 24 | Probiotic LB= *Lactobacillus bulgaricus* (1x10^9^ CFU/ml); n=24  Probiotic LC= *Lactobacillus casei Qian* (1x10^8^ CFU/ml); n=24  Probiotic LC= *Lactobacillus casei Qian* (1x10^9^ CFU/ml); n=24  Drug group= bisacodyl ; 100 mg/kg; n= 24 | 9 days | ↓ GI transit time and ↑ Fecal water content on 7-9 days (in all probiotic groups) |
| Zhao 2018 (30) | Experimental trial | 100; all females | mice (Kunming);  7 weeks old; loperamide-induced constipation | non-constipated group= standard diet,  constipated induced group= standard diet, | Probiotic LB= *Lactobacillus bulgaricus;* (1x10^9^ CFU/kg),  Probiotic LP-YS3 _low-dose_= *Lactobacillus plantarum* YS-3; (1x· 10^8^ CFU/kg)  Probiotic LP-YS3 _high-dose_ *Lactobacillus plantarum* YS-3; (1x 10^9^ CFU/kg), | 17 days | ↑ Number of stools; ↓ GI transit time (in all probiotic groups)  ↑ Fecal water content on 15-17 days (in all probiotic groups) |
| **HUMAN STUDIES** | | | | | | | |
| An 2010 (31) | Quasi-experimental | 19; 11 females | all: 77.1 ± 10.1 years  Chronic Constipation | non-control group; | *Lactobacillus acidophilus* (LH) CBT, *Pediococcus pentosaceus* (PP) CBT, *Bifidobacterium longum* SPM 1205; 3.0 × 10^11^ CFU/g | 14 days | No significant differences on stool consistency, on defecation frequency. |
| Anzawa 2019 (32) | RCT | 60; 43 females | all: 45.0 ± 8.9 years  Tendency for constipation (3–5 days/ week) | fermented milk; n= 20 | Probiotic: *Bifidobacterium lactis* GCL2505 (1 × 10^10^ CFU/100g)  Synbiotic**:** inulin (2.0 g/100 g) and *Bifidobacterium lactis* GCL2505 (1 × 10^10^ CFU/100g); fermented milk | 14 days | ↑ defecation frequency (in pro- and synbiotic groups) |
| Araújo 2021 (33) | RCT | 45; 40 females | Control: 30.95 ± 11.10 years; Probiotic:26.40 ± 8.47 years.  Functional constipation by Rome IV criteria | maltodextrin;  n= 20 | *Lactobacillus acidophilus* (NCFM), *Lactobacillus casei* (Lc-11),  *Lactococcus lactis* (Ll-23), *Bifidobacterium* *lactis* (HN019), and *Bifidobacterium bifidum* (Bb-06); 5x10^9^ CFU; capsules; 30 min after the last meal of the day | 30 days | No significant differences on stool consistency, on defecation frequency and Rome IV criteria |
| Botelho 2020 (34) | RCT | 5; 30 females | Control: 31.00 ± 11.64 years; Probiotic:71.0 ± 7.22 years  Functional constipation by Rome IV criteria | maltodextrin; n=14 | *Lactobacillus acidophilus* (NCFM), *Lactobacillus casei* (Lc-11),  *Lactococcus lactis* (Ll-23), *Bifidobacterium* *lactis* (HN019), and *Bifidobacterium* *bifidum* (Bb-06); 5x10^9^ CFU; capsules; 30 min after the last meal of the day | 30 days | No significant differences on stool consistency, on defecation frequency and Rome IV criteria |
| Chen 2019 (35) | Open-label | 62; 54 females | Hard stool: 27.5 (10.8). Normal Stool: 24.0 (7) years. Soft Stool: 23.0 (3.8) years  Functional constipation by Rome III criteria | non-control group; | *Lactobacillus casei Shirota*; 1×10^10^ CFU/100mL; fermented milk | 28 days | ↓ Painful effort during defecation, ↓ Feeling of incomplete evacuation, ↓ Straining during defecation, ↓ Abdominal discomfort, ↑ Stool consistency and defecation frequency  No significant difference in unsuccessful defecation attempt |
| Dimidi 2018 (36) | RCT | 75; 69 females | Control: 31 ± 10 years; Probiotic: 35 ± 12 years  Functional constipation by Rome III criteria | maltodextrin; n=38 | *Bifidobacterium* *lactis* NCC2818; 1.5 × 10^10^ CFU/d; powder; dissolved in water | 28 days | No significant differences on bloating, on gastrointestinal symptoms score (PAC-SYM), stool consistency, defecation frequency or CTT. |
| Fravetto 2013 (37) | RCT not-blind | 30; all females | Control: 40.8 ± 12.8 years; Probiotic: 37.5 ± 14.4  Functional constipation by Rome III criteria | Cheese;  n= 15 | *Bifidobacterium lactis* Bi-07; 1x10^8^ CFU/30g; cheese; preferably at breakfast | 30 days | ↑ Defecation Frequency |
| Fuyuki 2021 (38) | Quasi-experimentall | 31; 20 females | all: 63.7 ± 11.8 years  Functional constipation by Rome IV criteria or already under treatment for chronic  constipation | non-control group; | *Bifidobacterium bifidum* G9-1 (BBG9-1); 1×10^6^ to 1×10^9^ CFU/g; 2 tablet/time; 3x/d; during meals | 56 days | ↑ Defecation frequency and quality of life  No significant differences on stool consistency. |
| Gotoh 2020 (39) | RCT | 27; all females | all: 30 to 60 years  frequency of defecation of 3–5 times/week | same capsules without probiotic; n=N/E | Probiotic_low-dose_: *Lactobacillus cremoris* FC 1 × 10^7^ CFU, 2 capsules/time; 1x/d  Probiotic_high-dose_: *Lactobacillus cremoris* FC; 2 × 10^7^ CFU; 2 capsules/time; 1x/d | 14 days | ↑ Defecation frequency (in high-dose group)  No significant differences on stool consistency (in both groups) |
| Higashikawa 2009 (40) | RCT three-blind | 68; 49 females | Group A: 37.3 ± 12.5 years;  Group B: 35.1 ± 11.6 years;  Group C: 33.0 ± 13.0 years;  with intestinal complains (constipation, diarrhea, bloating) | non-control group; | Group A: plant-derived LAB *Lactobacillus* *plantarum* SN35N and SN13T at 95% and 5%, respectively);  Group B: plant-derived LAB (*Lactobacillus* *plantarum* SN13T and SN35N at 98% and 2%, respectively);  Group C: by animal-derived LAB (with *Lactococcus lactis* A6, *Streptococcus* (S.) *thermophilus* 510, and *Lactobacillus bulgaricus* C6 at 86.1%, 13.8%, and 0.1%, respectively).  All: > 2 x 10^8^ LAB/g; yogurt; 1x/d | 42 days | ↓ Abdominal pain, feeling of incomplete evacuation* (in all groups) |
| Ibarra 2018 (41) | RCT | 228; 182 females | All: 41.7 ± 14.0 years; functional constipation per Rome III criteria. | Microcrystalline cellulose and potato maltodextrin;  n= 76 | Probiotic _low-dose_: *Bifidobacterium animalis* subsp. *lactis* HN019; 1 x 10^9^ CFU  Probiotic _high-dose_: *Bifidobacterium animalis* subsp. *lactis* HN019; 1 x 10^10^ CFU  All: capsules; dissolved in dairy products | 28 days | ↑ Defecation frequency in participants with ≤ 3 times/week (subgroup analysis) (in both low and high-dose groups), ↓ straining during defecation (in high-dose group)  No significant differences on bloating, abdominal pain, PAC-SYM score; stool consistency; defecation frequency; colonic transit time. |
| Kang 2021 (42) | RCT | 80; 70 females | Control: 45.3 ± 1.8 years;  Probiotic: 44.4 ± 2.2 years; functional constipation per Rome III criteria. | Maltodextrin;  n= 40 | *Bifidobacterium coagulans* SNZ 1969; 1 × 10^9^ CFU; capsules; dissolved in water; | 56 days | ↑ CTT  ↓ Bowel discomfort symptom (BDS) score. Only after 6 weeks  ↑ Defecation frequency (after 2-wks) |
| Kim 2015 (43) | Quasi-experimental | 60; 39 females | Control: 32.0 ± 3.0 years;  Probiotic: 35.0 ± 5.0 years; functional constipation per Rome III criteria. | Non-constipated as controls, n= 30 | VSL#3 (*Bifidobacterium longum, Bifidobacterium infantis* and *Bifidobacterium breve*); *Lactobacillus acidophilus, Lactobacillus casei, Lactobacillus bulgaricus,* and *Lactobacillus plantarum*) and *Streptococcus thermophilus*); 4.5 x10^11^ CFU; sachet; 2x/d | 14 days | ↑ Stool consistency*  No significant differences on defecation frequency; |
| Koebnick 2003 (44) | RCT | 70; 38 females | Control: 44.6 ± 9.7 years;  Probiotic: 43.3±6.9 years;  With functional constipation | Beverage;  n= 35 | *Lactobacillus casei* Shirota (LcS); 6.5×10^9^ CFU; beverage | 28 days | ↓ Hard stools;  ↓ Flatulence  ↑ Stool consistency  ↑ Defecation frequency  No significant differences on bloating |
| Madempudi 2019 (45) | RCT | 100; 41 females | All: 43.92 ± 11.74 years;  functional constipation per Rome III criteria. | N/E;  n= 50 | *Bifidobacterium coagulans*; 2 × 10^9^ CFU; cápsules. | 28 days | ↑ Defecation frequency;  ↓ Pain during defecation;*  ↓ Abdominal pain; *  ↑ Stool consistency; |
| Martoni 2019 (46) | RCT | 94; 71 females | Control: 42.9 ± 13.8 years  Probiotic: 44.0 ± 11.3 years;  functional constipation per Rome III criteria. | rice maltodextrin;  n= 46 | *Lactobacillus acidophilus* DDS-1, *Bifidobacterium* *animalis* subsp. *lactis* UABla-12, *Bifidobacterium longum* UABl-14 and *Bifidobacterium* *bifidum* UABb-10; 1.5 × 10^10^; capsules; before or during meals | 28 days | ↑ Stool consistency (after 1-wk)  ↓ PAC-SYM score* |
| Mazlyn 2013 (47) | RCT | 90; 78 females | All: 31.7 ± 9.4 years;  with functional constipation per Rome III criteria. | fermented milk; n= 43 | *Lactobacillus casei* Shirota (LcS); 3.0 x 10^10^ CFU; 1x/day; fermented milk. | 28 days | ↓ Severity of constipation (tendency)  No significant differences on stool consistency, defecation frequency |
| Moreira 2017 (48) | RCT | 49; all females | All: 29.0 ± 7.98 years;  with functional constipation per Rome III criteria. | milk beverage; n= 24 | *Bifidobacterium animalis*; 3.2x10^7^ CFU; 1x/day; fermented milk; before or during breakfast. | 60 days | No significant differences on lumpy or hard stools; stool consistency; |
| Ou 2019 (49) | Quasi-experimental | 38; 36 females | All: 35.0 ± 12.0 years;  with functional constipation per Rome III criteria. | non-constipated as controls;  n= 22 | *Lactobacillus casei* Shirota (LcS); 1 × 10^10^ CFU; 1x/day; fermented dairy beverage; after their daily lunch. | 28 days | ↓ Pain during defecation; Incomplete feeling during defecation; Straining during defecation; Abdominal discomfort.  ↑ Defecation frequency;  No significant differences on stool consistency; |
| Pinheiro 2017 (50) | RCT | 80; 67 females | Control: 45 (21-65) years;  Probiotic: 50 (20-69) years;  with reduced bowel movements and other symptoms of GI discomfort | maltodextrin; n=40 | *Saccharomyces cerevisiae*; 500 mg; 1x/day; capsule. | 42 days | ↓ Bloating/distention; feeling of fullness; General GI discomfort (on subjects reporting milder)  ↑ Stool Consistency  No significant differences on defecation frequency; |
| Riezzo 2012 (51) | RCT-crossover | 20; 17 females | All: 38.8 ± 14.4 years;  with functional constipation per Rome III criteria. | artichokes; n=10 | *Lactobacillus paracasei* IMPC; 2 x 10^10^ CFU; 1x/day; artichokes. | 15 days | ↓ GSRS constipation cluster score; hard stool; feeling  of incomplete evacuation*  ↑ Stool Consistency*  ↑ Defecation Frequency |
| Riezzo 2017 (52) | RCT | 56; 52 females | All: 43.8±11.5 years;  with functional constipation per Rome III criteria. | tablets ≤2,000 LR cfu; n=28 | *Lactobacillus reuteri* DSM 17938 (LR DSM 17938); 7×10^8^ CFU; 2x/day; tablet; between meals. | 105 days | ↓ CSS total score*  No significant differences on stool consistency; defecation frequency; CTT; |
| Sakai 2011 (53) | Open-label | 39; 23 females | Control: 32.1 ± 13.6 years;  Probiotic: 35.4 ± 14.2 years;  BSF score < 3.0. | nothing;  n=20 | *Lactobacillus casei* YIT 9029; 6.5 x 10^9^; 1x/day; fermented milk. | 21 days | ↑ Stool Consistency; ↑ Defecation frequency  No significant differences on GI symptoms; |
| Waller 2011 (54) | RCT-triple blind | 88; 56 females | Control: 45.0 ± 11.0 years;  Probiotic_low dose_: 43 ± 12 years;  Probiotic_high dose_: 44 ± 11 years;  self-report of stool type 2–4 on the BSF; and average of 1–3 bowel movements/week. | rice maltodextrin; n=29 | Probiotic _low-dose_: *Bifidobacterium* *lactis* HN019 (1.8 x10^9^ CFU);  Probiotic _high-dose_: *Bifidobacterium lactis* HN019 (17.2 x 10^9^ CFU); 1x/day; capsule; with breakfast; dissolved in yogurt. | 14 days | ↓ Abdominal pain, ↓ Constipation, ↓ CTT (in both low and high dose groups)  ↓ Flatulence (high-dose) |
| Wang 2020 (55) | Open-label | 26; 21 females | All: 28.50 ± 5.52functional constipation per Rome III criteria. | non-control group; | *Lactobacillus bulgaricus* ND02, *Streptococcus thermophilus* ND03, *Lactobacillus casei* and *Bifidobacterium animalis ssp. lactis* V9; *Lactobacillus bulgaricus* ND02 (1 × 10^3^ CFU/mL), *Streptococcus thermophilus* ND03 (1 × 10^6^ CFU/mL), *Lactobacillus casei* Zhang (1.0 × 10^5^ CFU/mL), and *Bifidobacterium* *animalis* ssp. *lactis* V9 (4.0 × 10^7^ CFU/mL) 200g; 1x/day; fermented milk. | 28 days | ↓ Pain during defecation;  ↓ Time of defecation;  ↓ Straining during defecation  ↑ Stool consistency  ↑ Defecation frequency  No significant differences on incomplete defecation. |
| Yang 2008 (56) | RCT | 126; all females | Control: 46.4 ± 6.7 years;  Probiotic: 46.4 ± 9.8 years;  < 3 bowel movements/week; increased stool hardness; non-organic constipation and habitual constipation. | acidified milk; n= 63 | *Bifidobacterium* *lactis* DN-173010; 1.25 × 10^10^; 1x/day. | 14 days | ↑ Stool consistency  ↑ Defecation Frequency |

↑, Increased or Improve; ↓, Decreased; †Double-blind placebo-control, unless otherwise stated; GI: Gastrointestinal; RCT: Randomized Controlled Trial. CTT: Colonic Transit Time. N/E: Not reported. KGM:konjac glucomannan; CFU: colony-forming unity; KOG:Konjac oligo-glucomannan. Wk: week.

**Supplementary Table 3.** Studies evaluating the effect of synbiotics on the gastrointestinal tract in animal models and human studies.

| **Reference** | **Study Design†** | **Sample (n; sex)** | **Participants Characteristics**  **(age; health condition)** | **Control** | **Intervention** | **Period of supplementation** | **Main**  **results** |
| --- | --- | --- | --- | --- | --- | --- | --- |
| **ANIMAL MODELS** | | | | | | | |
| Lu 2021 (57) | Experimental trial | 80; all females | mice (BALB/c);  6 weeks old; loperamide-induced constipation | non-constipated group: standard diet; n= 8  constipated induced group: standard diet; n= 8 | Probiotic PP + F1-7= *Prunus persica* (PP) 5mL *+ Bifidobacterium animalis* F1-7; (2x10^8^ CFU); n= 8  Probiotic PP + FWDG= PP 5mL + *Lactobacillus plantarum* FWDG; (2x10^8^ CFU); n= 8  Probiotic PP + F34-3= PP 5mL + *Lactobacillus paracasei* F34-3; (2x10^8^ CFU); n= 8  Probiotic KGM+ F1-7: Konjac glucomannan (KGM) (5mL) + *Bifidobacterium animalis* F1-7 (2x10^8^ CFU); n= 8  Probiotic KGM + FWDG= KGM 5mL *+ Lactobacillus plantarum;* (2x10^8^ CFU); n= 8  Probiotic KGM + F34-3= KGM 5mL *+ Lactobacillus paracasei* F34-3; (2x10^8^ CFU); n= 8  Probiotic LGG*= Lactobacillus rhamnosus* GG; (2x10^8^ CFU); n= 8  Drug group= prucalopride succinate; n= 8 | N/E | ↓ GI transit time, ↑ Fecal water content (in all probiotic groups |
| Lu 2021 (58) | Experimental trial | 132; N/E | Zebrafish larvae (Wild type AB series);  4 months; loperamide-induced constipation | non-constipated group: standard diet; n=12  constipated induced group: standard diet; n=12  constipated induced group 2: standard diet + KGM, n=12  constipated induced group 2: standard diet + X11, n=12  constipated induced group 2: standard diet + KGM+X11, n=12 | Synbiotic KGM+X11= konjac glucomannan (KGM) + *Lactobacillus paracasei* X11; 1 × 10^8^ CFU; n=12  Synbiotic PP+X11= Prunus persica (PP) + *Lactobacillus paracasei* X11; 1 × 10^8^ CFU; n=12  Synbiotic PP+ YRL577= PP + *Lactobacillus casei* YRL577; 1 × 108CFU; n=12  Synbiotic KGM+ YRL577= *Lactobacillus casei* YRL577; 1 × 108CFU; n=12  Synbiotic KGM +BB12= KGM + *Bifidobacterium animalis* subsp*. lactis* BB12; 1 × 108CFU; n=12  Synbiotic PP+BB12= PP + *Bifidobacterium animalis* subsp*. lactis* BB12; 1 × 108CFU; n=12 | N/E | ↑ Intestinal peristalsis (in all groups, with better effect in KGM + X11 group) |
| **HUMAN STUDIES** | | | | | | | |
| Bazzocchi 2014 (59) | RCT | 42; 36 females | Control: 42.21 ± 12.88; Prebiotic: 45.09 ± 15.09 years  Functional constipation by Rome III criteria | maltodextrin;  n= 12 | Psyllogel Megafermenti: Psyllium fiber + five probiotic strains, belonging to different *Lactobacillus* and *Bifidobacterium* species; 2 bags/d. | 56 days | ↓ CTT |
| Cudmore 2016 (60) | RCT | 69; 64 females | Control: 42.21 ± 12.88; Prebiotic: 45.09 ± 15.09 years  Functional constipation by Rome III criteria | fine rice starch;  n= 34 | Psyllium husks (3.45g) + inulin (1.5g) + *Lactobacillus rhamnosus* PXN 54 (NCIMB 30188), *Bifidobacterium bifidum* PXN 23 (NCIMB 30179), *Lactobacillus acidophilus* PXN 35 (NCIMB 30184), *Lactobacillus plantarum* PXN 47 (NCIMB 30187) and *Lactobacillus bulgaricus* PXN 39 (NCIMB 30186) (6 x10^8^ CFU/strains combine); 2x/d; sachet; 30 min before a main meal; dissolved in water. | 28 days | ↓ Laxative use  No significant difference on PAC-SYM score |
| Ding 2016 (61) | RCT | 93; females | Control: 42.21 ± 12.88; Prebiotic: 45.09 ± 15.09 years  Functional constipation or IBS-C by Rome III criteria | maltodextrin; n= 45 | BIFICOPEC: Pectin (8g) + *Enterococci, Bifidobacteria,* and *Lactobacilli* (0,63g/strains combine); 2x/d; Not reported. | 84 days | ↑ Stool Consistency  ↑ Defecation Frequency  ↓ CTT  No significant difference on PAC-SYM score |
| Fateh 2011 (62) | RCT | 60; all males | Control: 42.21 ± 12.88; Prebiotic: 45.09 ± 15.09 years  Functional constipation by Rome III criteria | Mg-stearate and maltodextrin; n= 29 | Protexin: FOS + *Bifidobacterium, Lactobacillus, Streptococcus* species (1x10^8^ CFU); 2x/d; capsules; after breakfast and after dinner. | 28 days | ↓Stomach cramps  ↑ Stool Consistency  ↑ Defecation Frequency  No significant difference on PAC-SYM score; Abdominal pain; Abdominal discomfort; Bloating |
| Lim 2018 (63) | RCT | 85; 73 females | Control: 27.5 ± 6.5 years;  Synbiotic: 29.5 ± 8.34 years;  with functional constipation by Rome III | maltodextrin; n= 42 | Inulin + *Lactobacillus plantarum* LP01, *Bifidobacterium lactis* BB12 (1x10^10^ CFU); 1x/day; sachet; during morning. | 84 days | No significant difference on PAC-SYM score; stool consistency; defecation frequency |
| Magro 2014 (64) | RCT | 47; 43 females | Control: 32.7 ± 7.3 years;  Synbiotic: 31.5 ± 7.1 years;  with chronic constipation | yogurt;  n= 21 | Polydextrose (3.6g) + *Lactobacillus acidophilus* NCFM® (ATCC 700396) and *Bifidobacterium lactis* HN019 (AGAL NM97/09513) (1x10^9^ CFU/strain); 1x/day; yogurt; during morning. | 14 days | ↓ CTT  No significant difference on defecation frequency |
| Malpeli 2012 (65) | RCT | 83; all females | All: 40.7 ± N/E years;  with functional constipation by Rome III criteria | yogurt; n= N/E | Inulin(0.625g) + *Bifidobacterium Iactis* BBI2*, Lactobacillus casei* CRL 431, (1 x 10^9^ to 1 x 10^10^ CFU/strain); 2x/day; yogurt. | 15 days | ↓ bloating  ↓ CTT  No significant difference on stool consistency |
| Minamida 2015 (66) | RCT | 137; 113 females | All: 51.5 ± N/E years;  with functional constipation by Rome III criteria | okara powder; n= 68 | Okara powder + *Bifidobacterium* *coagulans* lilac-01 (1 × 10^8^ CFU/d); powder; 1x/day; food; . | 14 days | ↑ Stool Consistency  ↑ Defecation frequency  No significant difference on GI symptoms; |
| De Paula 2008 (67) | Open label, control trial | 266; all females | All: 34.9 ± 8.9 years;  functional constipation by Rome II criteria | yogurt; n= N/E | Inulin (0.5%) + *Bifidobacterium animalis* (DN-173010) (1x10^8^ CFU/g); 2x/day; yogurt. | 14 days | ↓ Straining during defecation  ↓ Pain during defecation  ↑ Stool consistency  ↑ Defecation frequency |
| Waitzberg 2013 (68) | RCT | 99; all females | All: 18 -75 years  functional constipation by Rome III criteria | maltodextrin; n= 50 | LACTOFOS: FOS (6g) + *Lactobacillus paracasei* (Lpc-37), *Lactobacillus rhamnosus* (HN001), *Lactobacillus acidophilus* (NCFM), *Bifidobacterium lactis* (HN019) (10^8^ - 10^9^ CFU); 2x/day; sachet; dissolved in water. | 30 days | ↑ Stool consistency  ↑ Defecation frequency  ↓ AGACHAN score  No significant difference on GI symptoms; |

↑, Increased or Improve; ↓, Decreased; †Double-blind placebo-control, unless otherwise stated; GI: Gastrointestinal; RCT : Randomized Controlled Trial. CTT: Colonic Transit Time. N/E: Not reported. CFU: colony-forming unit; FOS: fructooligosaccharides; GOS: galacto-oligosaccharides; AGACHAN: Constipation Scoring System. KGM: konjac glucomannan

**REFERENCE**

1. Han SH, Hong KB, Kim EY, Ahn SH, Suh HJ. Effect of dual-type oligosaccharides on constipation in loperamide-treated rats. *Nutr Res Pract* (2016) **10**:583–589. doi: 10.4162/nrp.2016.10.6.583

2. Lan J, Wang K, Chen G, Cao G, Yang C. Effects of inulin and isomalto-oligosaccharide on diphenoxylate-induced constipation, gastrointestinal motility-related hormones, short-chain fatty acids, and the intestinal flora in rats. *Food Funct* (2020) **11**:9216–9225. doi: 10.1039/d0fo00865f

3. Liang Y-X, Wen P, Wang Y, OuYang D-M, Wang D, Chen Y-Z, Song Y, Deng J, Sun Y-M, Wang H. The Constipation-Relieving Property of d-Tagatose by Modulating the Composition of Gut Microbiota. *Int J Mol Sci* (2019) **20**: doi: 10.3390/ijms20225721

4. Lu W-D, Wu M-L, Zhang J-X, Huang T-T, Du S-S, Cao Y-X. The effect of sodium carboxymethyl starch with high degree of substitution on defecation. *PLoS One* (2021) **16**:e0257012. doi: 10.1371/journal.pone.0257012

5. Su H, Chen J, Miao S, Deng K, Liu J, Zeng S, Zheng B, Lu X. Lotus seed oligosaccharides at various dosages with prebiotic activity regulate gut microbiota and relieve constipation in mice. *Food Chem Toxicol an Int J Publ Br Ind Biol Res Assoc* (2019) **134**:110838. doi: 10.1016/j.fct.2019.110838

6. Zhang X, Zheng J, Jiang N, Sun G, Bao X, Kong M, Cheng X, Lin A, Liu H. Modulation of gut microbiota and intestinal metabolites by lactulose improves loperamide-induced constipation in mice. *Eur J Pharm Sci* (2021) **158**:105676. doi: 10.1016/j.ejps.2020.105676

7. Bouhnik Y, Neut C, Raskine L, Michel C, Riottot M, Andrieux C, Guillemot F, Dyard F, Flourié B. Prospective, randomized, parallel-group trial to evaluate the effects of lactulose and polyethylene glycol-4000 on colonic flora in chronic idiopathic constipation. *Aliment Pharmacol Ther* (2004) **19**:889–899. doi: 10.1111/j.1365-2036.2004.01918.x

8. Chu JR, Kang S-Y, Kim S-E, Lee S-J, Lee Y-C, Sung M-K. Prebiotic UG1601 mitigates constipation-related events in association with gut microbiota: A randomized placebo-controlled intervention study. *World J Gastroenterol* (2019) **25**:6129–6144. doi: 10.3748/wjg.v25.i40.6129

9. Glibowski, P., Skrzypek, M., Ćwiklińska, M., Drozd, M., & Kowalska A. Chemical stability of fructans in apple beverages and their influence on chronic constipation. *Food Funct* (2020)0–21. doi: 10.1039/C9FO02596K.Food

10. Micka A, Siepelmeyer A, Holz A, Theis S, Schön C. Effect of consumption of chicory inulin on bowel function in healthy subjects with constipation: a randomized, double-blind, placebo-controlled trial. *Int J Food Sci Nutr* (2017) **68**:82–89. doi: 10.1080/09637486.2016.1212819

11. Müller M, Hermes GDA, Emanuel E C, Holst JJ, Zoetendal EG, Smidt H, Troost F, Schaap FG, Damink SO, Jocken JWE, et al. Effect of wheat bran derived prebiotic supplementation on gastrointestinal transit, gut microbiota, and metabolic health: a randomized controlled trial in healthy adults with a slow gut transit. *Gut Microbes* (2020) **12**: doi: 10.1080/19490976.2019.1704141

12. Rasmussen HE, Hamaker B, Rajan KB, Mutlu E, Green SJ, Brown M, Kaur A, Keshavarzian A, Lafayette W, Facility S. Starch-entrapped microsphere fibers improve bowel habit but do not exhibit prebiotic capacity in those with unsatisfactory bowel habits: a Phase I, randomized, double-blind, controlled human trial. *Nutr Res* (2018) **44**:27–37. doi: 10.1016/j.nutres.2017.05.015.Starch-entrapped

13. Vandeputte D, Falony G, Vieira-Silva S, Wang J, Sailer M, Theis S, Verbeke K, Raes J. Prebiotic inulin-type fructans induce specific changes in the human gut microbiota. *Gut* (2017) **66**:1968–1974. doi: 10.1136/gutjnl-2016-313271

14. Deng Y, Li M, Mei L, Cong LM, Liu Y, Zhang BB, He CY, Zheng PY, Yuan JL. Manipulation of intestinal dysbiosis by a bacterial mixture ameliorates loperamide-induced constipation in rats. *Benef Microbes* (2018) **9**:453–464. doi: 10.3920/BM2017.0062

15. Eor JY, Tan PL, Lim SM, Choi DH, Yoon SM, Yang SY, Kim SH. Laxative effect of probiotic chocolate on loperamide-induced constipation in rats. *Food Res Int* (2019) **116**:1173–1182. doi: 10.1016/j.foodres.2018.09.062

16. Gan Y, Liang J, Diao W, Zhou X, Mu J, Pang L, Tan F, Zhao X. Lactobacillus plantarum KSFY06 and geniposide counteract montmorillonite-induced constipation in Kunming mice. *Food Sci Nutr* (2020) **8**:5128–5137. doi: 10.1002/fsn3.1814

17. Hayeeawaema F, Wichienchot S, Khuituan P. Amelioration of gut dysbiosis and gastrointestinal motility by konjac oligo-glucomannan on loperamide-induced constipation in mice. *Nutrition* (2020) **73**:110715. doi: 10.1016/j.nut.2019.110715

18. Kim MG, Jo K, Cho K, Park SS, Suh HJ, Hong K-B. Prebiotics/Probiotics Mixture Induced Changes in Cecal Microbiome and Intestinal Morphology Alleviated the Loperamide-Induced Constipation in Rat. *Food Sci Anim Resour* (2021) **41**:527–541. doi: 10.5851/kosfa.2021.e17

19. Lee CS, Tan PL, Eor JY, Choi DH, Park M, Seo SK, Yoon S, Yang S, Kim SH. Prophylactic use of probiotic chocolate modulates intestinal physiological functions in constipated rats. *J Sci Food Agric* (2019) **99**:3045–3056. doi: 10.1002/jsfa.9518

20. Li C, Nie S-P, Zhu K-X, Xiong T, Li C, Gong J, Xie M-Y. Effect of Lactobacillus plantarum NCU116 on loperamide-induced constipation in mice. *Int J Food Sci Nutr* (2015) **66**:533–538. doi: 10.3109/09637486.2015.1024204

21. Lu Y, Zhang J, Yi H, Zhang Z, Zhang L. Screening of intestinal peristalsis-promoting probiotics based on a zebrafish model. *Food Funct* (2019) **10**:2075–2082. doi: 10.1039/c8fo02523a

22. Makizaki Y, Uemoto T, Yokota H, Yamamoto M, Tanaka Y, Ohno H. Improvement of loperamide-induced slow transit constipation by Bifidobacterium bifidum G9-1 is mediated by the correction of butyrate production and neurotransmitter profile due to improvement in dysbiosis. *PLoS One* (2021) **16**:e0248584. doi: 10.1371/journal.pone.0248584

23. Tan Q, Hu J, Zhou Y, Wan Y, Zhang C, Liu X, Long X, Tan F, Zhao X. Inhibitory Effect of Lactococcus lactis subsp. lactis HFY14 on Diphenoxylate-Induced Constipation in Mice by Regulating the VIP-cAMP-PKA-AQP3 Signaling Pathway. *Drug Des Devel Ther* (2021) **15**:1971–1980. doi: 10.2147/DDDT.S309675

24. Wang L, Hu L, Xu Q, Yin B, Fang D, Wang G, Zhao J, Zhang H CW. Bifidobacterium adolescentis exerts strain-specific effects on constipation induced by loperamide in BALB/c mice. *Int J Mol Sci* (2017) **18**:318. doi: 10.3390/ijms18020318

25. Wang L, Chen C, Cui S, Lee Y-K, Wang G, Zhao J, Zhang H, Chen W. Adhesive Bifidobacterium Induced Changes in Cecal Microbiome Alleviated Constipation in Mice. *Front Microbiol* (2019) **10**:1721. doi: 10.3389/fmicb.2019.01721

26. Wang R, Sun J, Li G, Zhang M, Niu T, Kang X, Zhao H, Chen J, Sun E, Li Y. Effect of Bifidobacterium animalis subsp. lactis MN-Gup on constipation and the composition of gut microbiota. *Benef Microbes* (2020) **12**:31–42. doi: 10.3920/BM2020.0023

27. Wang G, Yang S, Sun S, Si Q, Wang L, Zhang Q, Wu G, Zhao J, Zhang H, Chen W. Lactobacillus rhamnosus Strains Relieve Loperamide-Induced Constipation via Different Pathways Independent of Short-Chain Fatty Acids. *Front Cell Infect Microbiol* (2020) **10**:423. doi: 10.3389/fcimb.2020.00423

28. Zhang J, Chen B, Liu B, Zhou X, Mu J, Wang Q, Zhao X, Yang Z. Preventive Effect of Lactobacillus fermentum CQPC03 on Activated Carbon-Induced Constipation in ICR Mice. *Medicina (Kaunas)* (2018) **54**: doi: 10.3390/medicina54050089

29. Zhao X, Suo H-Y, Qian Y, Li G-J, Liu Z-H, Li J. Therapeutic effects of Lactobacillus casei Qian treatment in activated carbon-induced constipated mice. *Mol Med Rep* (2015) **12**:3191–3199. doi: 10.3892/mmr.2015.3737

30. Zhao X, Yi R, Qian Y, Park KY. Lactobacillus plantarum YS-3 Prevents Activated Carbon-Induced Constipation in Mice. *J Med Food* (2018) **21**:575–584. doi: 10.1089/jmf.2017.4109

31. An HM, Baek EH, Jang S, Lee DK, Kim MJ, Kim JR, Lee KO, Park JG, Ha NJ. Efficacy of Lactic Acid Bacteria (LAB) supplement in management of constipation among nursing home residents. *Nutr J* (2010) **9**:5. doi: 10.1186/1475-2891-9-5

32. Anzawa D, Mawatari T, Tanaka Y, Yamamoto M, Genda T, Takahashi S, Nishijima T, Kamasaka H, Suzuki S, Kuriki T. Effects of synbiotics containing Bifidobacterium animalis subsp. lactis GCL2505 and inulin on intestinal bifidobacteria: A randomized, placebo-controlled, crossover study. *Food Sci Nutr* (2019) **7**:1828–1837. doi: 10.1002/fsn3.1033

33. Araújo A de M, Botelho PB, Ribeiro DJS, Magalhães KG, Nakano EY, Arruda SF. A multiple-strain probiotic product provides a better enzymatic antioxidant response in individuals with constipation in a double-blind randomized controlled trial. *Nutrition* (2021) **89**: doi: 10.1016/j.nut.2021.111225

34. Botelho PB, Ferreira MVR, Araújo A de M, Mendes MM, Nakano EY. Effect of multispecies probiotic on gut microbiota composition in individuals with intestinal constipation: A double-blind, placebo-controlled randomized trial. *Nutrition* (2020) **78**: doi: 10.1016/j.nut.2020.110890

35. Chen S, Ou Y, Zhao L, Li Y, Qiao Z, Hao Y, Ren F. Differential effects of lactobacillus casei strain shirota on patients with constipation regarding stool consistency in China. *J Neurogastroenterol Motil* (2019) **25**:148–158. doi: 10.5056/jnm17085

36. Dimidi E, Zdanaviciene A, Christodoulides S, Taheri S, Louis P, Duncan PI, Emami N, Crabbé R, De Castro CA, McLean P, et al. Randomised clinical trial: Bifidobacterium lactis NCC2818 probiotic vs placebo, and impact on gut transit time, symptoms, and gut microbiology in chronic constipation. *Aliment Pharmacol Ther* (2019) **49**:251–264. doi: 10.1111/apt.15073

37. Favretto DC, Pontin B, Moreira TR. Efeito da ingestão de um queijo acrescido de microrganismos probióticos (Bifidobacterium lactis Bi-07) na melhora de sintomas de constipação. *Arq Gastroenterol* (2013) **50**:196–201. doi: 10.1590/S0004-28032013000200035

38. Fuyuki A, Higurashi T, Kessoku T, Ashikari K, Yoshihara T, Misawa N, Iwaki M, Kobayashi T, Ohkubo H, Yoneda M, et al. Efficacy of Bifidobacterium bifidum G9-1 in improving quality of life in patients with chronic constipation: a prospective intervention study. *Biosci microbiota, food Heal* (2021) **40**:105–114. doi: 10.12938/bmfh.2020-073

39. Gotoh Y, Nanba F, Shioya N, Sugimura H, Suzuki T. A dose-finding study for a supplement containing Lactococcus lactis subsp. cremoris FC in healthy adults with mild constipation. *Biosci microbiota, food Heal* (2020) **39**:19–22. doi: 10.12938/bmfh.19-009

40. Higashikawa F, Noda M, Awaya T, Nomura K, Oku H, Sugiyama M. Improvement of constipation and liver function by plant-derived lactic acid bacteria: A double-blind, randomized trial. *Nutrition* (2010) **26**:367–374. doi: http://dx.doi.org/10.1016/j.nut.2009.05.008

41. Ibarra A, Latreille-Barbier M, Donazzolo Y, Pelletier X, Ouwehand AC. Effects of 28-day Bifidobacterium animalis subsp. lactis HN019 supplementation on colonic transit time and gastrointestinal symptoms in adults with functional constipation: A double-blind, randomized, placebo-controlled, and dose-ranging trial. *Gut Microbes* (2018) **9**:236–251. doi: 10.1080/19490976.2017.1412908

42. Kang S, Park MY, Brooks I, Lee J, Kim SH, Kim JY, Oh B, Kim JW, Kwon O. Spore-forming Bacillus coagulans SNZ 1969 improved intestinal motility and constipation perception mediated by microbial alterations in healthy adults with mild intermittent constipation: A randomized controlled trial. *Food Res Int* (2021) **146**:110428.

43. Kim SE, Choi SC, Park KS, Park MI, Shin JE, Lee TH, Jung KW, Koo HS, Myung SJ. Change of fecal flora and effectiveness of the short-term VSL#3 probiotic treatment in patients with functional constipation. *J Neurogastroenterol Motil* (2015) **21**:111–120. doi: 10.5056/jnm14048

44. Koebnick C, Wagner I, Leitzmann P, Stern U, Zunft HJF. Probiotic beverage containing Lactobacillus casei Shirota improves gastrointestinal symptoms in patients with chronic constipation. *Can J Gastroenterol* (2003) **17**:655–659. doi: 10.1155/2003/654907

45. Madempudi RS, Neelamraju J, Ahire JJ, Gupta SK, Shukla VK. Bacillus coagulans Unique IS2 in Constipation: A Double-Blind, Placebo-Controlled Study. *Probiotics Antimicrob Proteins* (2020) **12**:335–342. doi: 10.1007/s12602-019-09542-9

46. Martoni CJ, Evans M, Chow C-ET, Chan LS, Leyer G, C.J. M, M. E, C.-E.T. C, L.S. C, G. L. Impact of a probiotic product on bowel habits and microbial profile in participants with functional constipation: A randomized controlled trial. *J Dig Dis* (2019) **20**:435–446. doi: 10.1111/1751-2980.12797

47. Mazlyn MM, Nagarajah LH-L, Fatimah A, Norimah AK, Goh K-L. Effects of a probiotic fermented milk on functional constipation: a randomized, double-blind, placebo-controlled study. *J Gastroenterol Hepatol* (2013) **28**:1141–1147. doi: 10.1111/jgh.12168

48. Moreira TR, Leonhardt D, Conde SR. A influência de bebida láctea com cultura probiótica (Bifidobacterium animalis) no tratamento dos sintomas de constipação. *Arq Gastroenterol* (2017) **54**:206–210. doi: 10.1590/s0004-2803.201700000-27

49. Ou Y, Chen S, Ren F, Zhang M, Ge S, Guo H, Zhang H, Zhao L. Lactobacillus casei Strain Shirota Alleviates Constipation in Adults by Increasing the Pipecolinic Acid Level in the Gut. *Front Microbiol* (2019) **10**:324. doi: 10.3389/fmicb.2019.00324

50. Pinheiro I, Robinson L, Verhelst A, Marzorati M, Winkens B, den Abbeele P Van, Possemiers S. A yeast fermentate improves gastrointestinal discomfort and constipation by modulation of the gut microbiome: results from a randomized double-blind placebo-controlled pilot trial. *BMC Complement Altern Med* (2017) **17**:441. doi: 10.1186/s12906-017-1948-0

51. Riezzo G, Orlando A, D’Attoma B, Guerra V, Valerio F, Lavermicocca P, De Candia S. Randomised clinical trial: Efficacy of Lactobacillus paracasei-enriched artichokes in the treatment of patients with functional constipation-a double-blind, controlled, crossover study. *Aliment Pharmacol Ther* (2012) **35**:441–450. doi: 10.1111/j.1365-2036.2011.04970.x

52. Riezzo G, Orlando A, D’Attoma B, Linsalata M, Martulli M, Russo F. Randomised double blind placebo controlled trial on Lactobacillus reuteri DSM 17938: Improvement in symptoms and bowel habit in functional constipation. *Benef Microbes* (2018) **9**:51–60. doi: 10.3920/BM2017.0049

53. Sakai T, Makino H, Ishikawa E, Oishi K, Kushiro A. Fermented milk containing Lactobacillus casei strain Shirota reduces incidence of hard or lumpy stools in healthy population. *Int J Food Sci Nutr* (2011) **62**:423–430. doi: 10.3109/09637486.2010.542408

54. Waller PA, Gopal PK, Leyer GJ, Ouwehand AC, Reifer C, Stewart ME, Miller LE. Dose-response effect of Bifidobacterium lactis HN019 on whole gut transit time and functional gastrointestinal symptoms in adults. *Scand J Gastroenterol* (2011) **46**:1057–1064. doi: 10.3109/00365521.2011.584895

55. Wang J, Bai X, Peng C, Yu Z, Li B, Zhang W, Sun Z, Zhang H. Fermented milk containing Lactobacillus casei Zhang and Bifidobacterium animalis ssp. lactis V9 alleviated constipation symptoms through regulation of intestinal microbiota, inflammation, and metabolic pathways. *J Dairy Sci* (2020) **103**:11025–11038. doi: 10.3168/jds.2020-18639

56. Yang Y-X, He M, Hu G, Wei J, Pages P, Yang X-H, Bourdu-Naturel S. Effect of a fermented milk containing Bifidobacterium lactis DN-173010 on Chinese constipated women. *World J Gastroenterol* (2008) **14**:6237–6243. doi: 10.3748/wjg.14.6237

57. Lu Y, Yu Z, Zhang Z, Liang X, Gong P, Yi H, Yang L, Liu T, Shi H, Zhang L. Bifidobacterium animalis F1-7 in combination with konjac glucomannan improves constipation in miceviahumoral transport. *Food Funct* (2021) **12**:791–801. doi: 10.1039/d0fo02227f

58. Lu Y, Zhang J, Zhou X, Guan M, Zhang Z, Liang X, Tong L, Yi H, Gong P, Bai L, et al. The edible Lactobacillus paracasei X11 with Konjac glucomannan promotes intestinal motility in zebrafish. *Neurogastroenterol Motil Off J Eur Gastrointest Motil Soc* (2021) **33**:e14196. doi: 10.1111/nmo.14196

59. Bazzocchi G, Giovannini T, Giussani C, Brigidi P, Turroni S. Effect of a new synbiotic supplement on symptoms, stool consistency, intestinal transit time and gut microbiota in patients with severe functional constipation: a pilot randomized double-blind, controlled trial. *Tech Coloproctol* (2014) **18**:945–953. doi: 10.1007/s10151-014-1201-5

60. Cudmore S, Doolan A, Lacey S, Shanahan F. A randomised, double-blind, placebo-controlled clinical study: the effects of a synbiotic, Lepicol, in adults with chronic, functional constipation. *Int J Food Sci Nutr* (2017) **68**:366–377. doi: 10.1080/09637486.2016.1244661

61. Ding C, Ge X, Zhang X, Tian H, Wang H, Gu L, Gong J, Zhu W, Li N. Efficacy of synbiotics in patients with slow transit constipation: A prospective randomized trial. *Nutrients* (2016) **8**:1–10. doi: 10.3390/nu8100605

62. Fateh R, Iravani S, Frootan M, Saadat S, Rasouli MR. Synbiotic preparation in men suffering from functional constipation: a randomised controlled trial. *Swiss Med Wkly* (2011) **141**:

63. Lim YJ, Jamaluddin R, Hazizi AS, Chieng JY. Effects of Synbiotics among Constipated Adults in Serdang, Selangor, Malaysia-A Randomised, Double-Blind, Placebo-Controlled Trial. *Nutrients* (2018) **10**: doi: 10.3390/nu10070824

64. Magro DO, de Oliveira LMR, Bernasconi I, Ruela M de S, Credidio L, Barcelos IK, Leal RF, Ayrizono M de LS, Fagundes JJ, Teixeira L de B, et al. Effect of yogurt containing polydextrose, Lactobacillus acidophilus NCFM and Bifidobacterium lactis HN019: a randomized, double-blind, controlled study in chronic constipation. *Nutr J* (2014) **13**:75. doi: 10.1186/1475-2891-13-75

65. Malpeli A, Gonzalez S, Vicentin D, Apas A, Gonzalez HF, A. M, S. G, D. V, A. A, H.F. G, et al. Randomised, double-blind and placebo-controlled study of the effect of a synbiotic dairy product on orocecal transit time in healthy adult women. *Nutr Hosp* (2012) **27**:1314–1319. doi: 10.3305/nh.2012.27.4.5770

66. Minamida K, Nishimura M, Miwa K, Nishihira J. Effects of dietary fiber with Bacillus coagulans lilac-01 on bowel movement and fecal properties of healthy volunteers with a tendency for constipation. *Biosci Biotechnol Biochem* (2015) **79**:300–306.

67. De Paula JA, Carmuega E, Weill R. Effect of the ingestion of a symbiotic yogurt on the bowel habits of women with functional constipation. *Acta Gastroenterol Latinoam* (2008) **38**:16–25.

68. Waitzberg DL, Logullo LC, Bittencourt AF, Torrinhas RS, Shiroma GM, Paulino NP, Teixeira-da-Silva ML. Effect of synbiotic in constipated adult women - A randomized, double-blind, placebo-controlled study of clinical response. *Clin Nutr* (2013) **32**:27–33. doi: 10.1016/j.clnu.2012.08.010
